# ER stress and the decline and fall of pancreatic beta cells in type 1 diabetes

**DOI:** 10.3109/03009734.2015.1135217

**Published:** 2016-02-18

**Authors:** Flora Brozzi, Décio L. Eizirik

**Affiliations:** ULB Center for Diabetes Research, Medical Faculty, Université Libre de Bruxelles (ULB), Brussels, Belgium

**Keywords:** Apoptosis, c-Jun N-terminal kinase, cytokines, ER stress, IRE1α, type 1 diabetes

## Abstract

Components of the unfolded protein response (UPR) modulate beta cell inflammation and death in early type 1 diabetes (T1D). The UPR is a mechanism by which cells react to the accumulation of misfolded proteins in the endoplasmic reticulum (ER). It aims to restore cellular homeostasis, but in case of chronic or overwhelming ER stress the persistent activation of the UPR triggers apoptosis, contributing to the loss of beta cells in both T1D and type 2 diabetes. It remains to be determined how and why the transition from ‘physiological’ to ‘pathological’ UPR takes place. A key component of the UPR is the ER transmembrane protein IRE1α (inositol-requiring enzyme 1α). IRE1α activity is modulated by both intra-ER signals and by the formation of protein complexes at its cytosolic domain. The amplitude and duration of IRE1α signaling is critical for the transition between the adaptive and cell death programs, with particular relevance for the activation of the pro-apoptotic c-Jun N-terminal kinase (JNK) in beta cells. In the present review we discuss the available information on IRE1α-regulating proteins in beta cells and their downstream targets, and the important differences observed between cytokine-induced UPR in human and rodent beta cells.

Free himself from envy and uncharitable speech, he would not suffer them in others … His works give evidence of careful writing … As he was ready to lay aside his own studies in order to help others, he was much loved and had many friends. (Jacob Burckhardt, *The Civilization of the Renaissance in Italy*, Phaidon Press Limited, London, 1995, pp 180–1)

The description above of the fifteenth-century humanist and scholar Pomponius Laetus serves also as an accurate description of Claes Hellerstrom’s leadership in the laboratory.

## Endoplasmic reticulum (ER) stress and the unfolded protein response (UPR) in pancreatic beta cells

Pancreatic beta cells are responsible for maintaining physiological glucose concentrations. For this purpose, they ‘sense’ glucose levels via their glucose metabolism and associated ATP generation, and respond to increases in glucose levels by synthesizing and releasing insulin. The newly transcribed insulin mRNA is translated at the endoplasmic reticulum (ER), and following nutrient stimulation there may be a 10-fold increase in insulin synthesis, representing 50% of the total protein synthesis by beta cells (1). The magnitude of the insulin synthesis and its fluctuations—inherent to the different amounts of food ingested and occasional fasting—poses a great challenge to the beta cell ER. To face this challenge, the beta cells, as other secretory cells, actively survey the state of protein folding inside the ER. This control is done by chaperones of the Hsp70 and Hsp40 families, chaperone-like proteins, as well as lectins (2–5). A key ER member of the Hsp70 family is the chaperone immunoglobulin heavy chain binding protein, BiP (also called GRP78) (2–4). When faced with excessive accumulation of misfolded proteins in the ER, which causes the so-called ER stress, beta cells respond by triggering the unfolded protein response (UPR). The UPR signaling is mediated via three main transmembrane sensors: endoribonuclease inositol-requiring protein 1α (IRE1α), protein kinase RNA-like endoplasmic reticulum kinase (PERK), and activating transcription factor 6 (ATF6) (6,7). Under basal condition BiP constitutively binds to the luminal domains of the three sensors, preventing their activation. When misfolded proteins accumulate in the ER, BiP dissociates from the UPR sensors and binds to the exposed hydrophobic domains of the unfolded proteins (8). This induces oligomerization and auto-transphosphorylation of IRE1α and PERK, with their consequent activation. At the same time, a conformational change in ATF6 exposes an ER export motif, and it translocates to the Golgi where it is cleaved. The cytosolic domain of ATF6 acts as a powerful transcription factor promoting adaptation (9). The activation of PERK induces eIF2α phosphorylation, leading to attenuation of global protein synthesis, thus reducing the load of unfolded proteins in the ER (10,11). Activating transcription factor 4 (ATF4) mRNA contains particular translation initiation elements that allow its translation (12), in spite of the global inhibition of protein synthesis, and generates a transcription factor controlling the expression of genes involved in protein folding, antioxidant response, autophagy, and apoptosis (13). In response to luminal activation, IRE1α dimerizes and trans-autophosphorylates, inducing a conformational change that activates its RNase domain. The main target of IRE1α’s endoribonuclease activity is X-box binding protein 1 (XBP1) mRNA: the excision of a 26-nucleotide intron in XBP1 mRNA produces a stable and active transcription factor known as XBP1 spliced (XBP1s) that controls expression of genes involved in protein folding, secretion, endoplasmic-reticulum-associated protein degradation (ERAD), and lipid synthesis (14–16). In addition, XBP1s may heterodimerize with ATF6 to control distinct gene expression patterns (17). IRE1α is also involved in the degradation of RNAs (known as regulated IRE1α-dependent decay or RIDD) (18), including ER-localized mRNAs (such as insulin in beta cells) (19), ribosomal RNA, and microRNAs, reducing the amount of proteins translated in the ER (20–22).

Previous reviews have described in detail the role of UPR in beta cell function and survival (6,23–26), including its role in the cross-talk with inflammatory pathways in type 1 diabetes (27) and in induction of apoptosis in type 2 diabetes (24,28,29). In the present article we will focus on two recently described aspects of the UPR in beta cells, namely the regulation of the crucial IRE1α/c-Jun N-terminal kinase (JNK) and IRE1α/XBP1s pathways and their role in beta cell apoptosis, and the important differences between the UPR in human and rodent beta cells.

## The transition from physiological to pro-apoptotic UPR

If the UPR does not succeed in alleviating the ER stress, the affected cells trigger the apoptotic program via the intrinsic or mitochondrial pathway of cell death (6,30). Of note, unresolved ER stress contributes to progressive beta cell death in both type 1 and type 2 diabetes (6,23,24,27) and in rodent models of these diseases (31,32). The persistent activation of the PERK/eIF2α/ATF4 pathway induces the transcription of C/EBP homologous protein (CHOP), which inhibits expression of the anti-apoptotic BCL-2 to promote cell death (6,24,33). Under prolonged ER stress, IRE1α forms a molecular complex with apoptosis signal-regulating kinase-1 (ASK1) and TNF receptor-associated factor 2 (TRAF2), triggering JNK phosphorylation (34), a crucial inducer of beta cell apoptosis (35–41). Moreover, XBP1 mRNA splicing progressively decreases (attenuating the pro-survival effects of the transcription factors) whereas PERK signaling is maintained, favoring the expression of downstream pro-apoptotic components (7,42). What ultimately determines the transition from ‘physiological’ to ‘apoptotic’ UPR remains, however, to be clarified (23). A recent study suggests that the timing of IRE1α and PERK signaling events, rather than a switch of activity from one UPR branch to another, is critical to determine cellular outcome (43). This observation highlights the complexity of the UPR and the importance of a fine-tuned regulation of the amplitude and kinetics of IRE1α and PERK activation. Accumulating evidence indicates that the changing nature of IRE1α signaling is critical for this transition in pancreatic beta cells (23,42,44). Particularly, and as discussed below, IRE1α-induced JNK activation seems to be necessary for cytokine-induced apoptosis in rat and human pancreatic beta cells (40,41).

## IRE1α—a crucial UPR sensor that responds to cues from both the ER and the cytosol

IRE1α activation is fine-tuned by the formation of molecular complexes both at its ER luminal and cytosolic regions. Besides the interaction with BiP, the luminal region of IRE1α can bind to the hydrophobic domains of unfolded proteins, being directly activated by them during ER stress (45). Furthermore, protein disulfide isomerase A6 (PDIA6) was recently shown to control activation of IRE1α signaling through a direct binding at its ER luminal region (46,47).

Fine-tuning regulation of IRE1α also takes place at its cytosolic domain (44). The concept of the ‘UPRosome’ predicts IRE1α as a scaffold where many components assemble selectively to regulate its activity (amplitude and kinetics) thus controlling specific downstream pathways (48,49). Interestingly, many regulators of IRE1α have relevant roles in apoptosis (44), including members of the BCL-2 family such as BAX, BAK (50), and the BH3-only proteins PUMA, BIM, and DP5 (38,51). Although the list of cytosolic IRE1α-binding partners is increasing (44), only few regulators have been identified so far in pancreatic beta cells.

IRE1α is differentially regulated during early beta cell adaptation to high glucose concentrations or in the course of severe ER stress (52). In this process, the formation of a glucose-induced ternary complex between IRE1α/RACK1/PP2A provides negative feedback on glucose stimulation of IRE1α through its dephosphorylation by PP2A. During persistent ER stress, however, the dissociation of PP2A from RACK1 contributes to sustained IRE1α phosphorylation, leading to XBP1 splicing, ERAD, and activation of JNK. These signals, together with the activation of other UPR pathways, cause beta cell dysfunction and eventually apoptosis (52).

**Figure 1.**
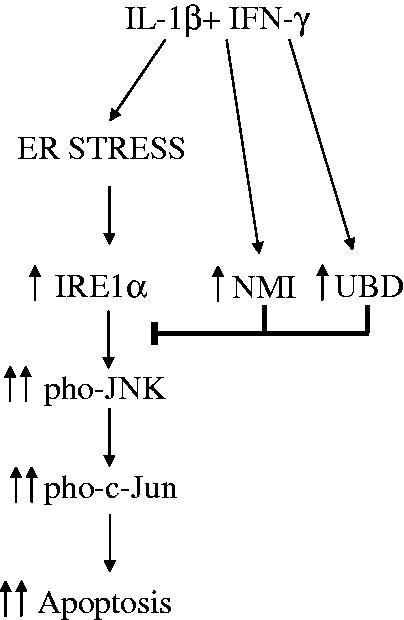
Role of the IRE1α-interacting proteins NMI and UBD in cytokine-induced beta cell apoptosis. The pro-inflammatory cytokines IL-1β + IFN-γ induce ER stress and activation of the IRE1α/JNK/c-Jun pathway that contributes to beta cell apoptosis. In parallel, IL-1β + IFN-γ induce the expression of NMI and UBD that bind to IRE1α and negatively regulate the phosphorylation of JNK, providing a negative feedback on this pro-apoptotic pathway. This feedback may prevent beta cell death in the context of mild and transitory local inflammation, but will not prevent beta cell death during a protracted autoimmune assault. The figure is based on findings from Brozzi F et al. (40) and Brozzi F et al., submitted for publication.

The combination of a high-throughput mammalian two-hybrid technology, MAPPIT (MAmmalian Protein-Protein Interaction Trap), with functional genomic analysis of human and rodent beta cells exposed to pro-inflammatory cytokines allowed us to identify two novel proteins that interact with IRE1α and are modified by pro-inflammatory cytokine exposure in pancreatic beta cells. Interestingly, these proteins, namely N-myc interactor (NMI) (40) and ubiquitin D (UBD) (Brozzi et al., submitted for publication), do not modify cytokine-induced IRE1α endoribonuclease activity as evaluated by XBP1 splicing and Ins-2 mRNA degradation (40) (Brozzi et al., submitted for publication). Their main role is to provide a negative feedback on IRE1α-induced activation of JNK. Indeed, inhibition of both NMI and UBD increases cytokine-induced JNK phosphorylation in beta cells after, respectively, 8 and 12 h or 2 and 8 h of exposure. Double knock-down (KD) of NMI and IRE1α, or UBD and IRE1α, reverse the up-regulation on JNK phosphorylation, confirming the regulatory function of NMI and UBD on IRE1α-dependent JNK activation in beta cells. Furthermore, double KD of NMI and JNK, or UBD and JNK, reverse the increase in beta cell apoptosis observed following individual KD of NMI or UBD, confirming the central role for the IRE1α-JNK pathway in cytokine-induced beta cell apoptosis (Figure 1) (40) (Brozzi et al., submitted for publication). Against this background, we hypothesize that during cytokine-induced UPR activation cytosolic proteins such as NMI and UBD modulate the induction of IRE1α, fine-tuning the signals provided by accumulation of misfolded proteins in the ER and delaying the triggering of apoptosis via JNK activation. This mechanism will prevent excessive beta cell apoptosis in case of limited innate immune response, but will not suffice to prevent cell death in the course of a protracted autoimmune assault and consequent long-term ER stress UPR activation.

## The UPR in human and rodent islets—different inducers and different pathways of responses

The ability of the long-lived beta cells to endure different assaults is a key component to prevent the development of diabetes (53). Human beta cells are more resistant than rat or mouse beta cells to cytotoxic agents such as sodium nitroprusside (a NO donor), streptozotocin (an alkylating agent), and alloxan (a generator of oxygen free radicals) (54–56). This may be due to the fact that human islet cells have constitutively higher expression of Hsp70, catalase, and superoxide dismutase (SOD) as compared to mouse and rat islets (55), probably as an adaptation to the much longer life span of human beta cells. It has been recently suggested that this high expression of Hsp70 in human islets is an indication of stressed cells secondary to poor islet isolation (57). This concept is, however, not correct. Thus, Hsp70 remains several folds higher in human than in rat islets even after 4 weeks *in vivo* following implantation into normoglycemic nude mice (55). Furthermore, the human islet preparations studied that showed high levels of Hsp70 showed also excellent insulin release in response to glucose (>5-fold increase comparing high versus low glucose concentrations), both *in vitro* and *in vivo*, confirming their healthy status (54,58–60).

Human islet cells also respond to chemical ER stressors differently compared to rat beta cells (61). Thus, the latter are particularly sensitive to ER stressors that block the sarcoendoplasmic reticulum calcium transport ATPase (SERCA)-2b, such as cyclopiazonic acid (CPA) and thapsigargin; these stressors increase expression of the ER stress markers XBP1s, CHOP, and BiP and lead to a higher level of apoptosis as compared to other ER stressors such as tunicamycin (a blocker of protein glycosylation in the ER (62)) and Brefeldin A (an inhibitor of vesicle transport between the ER and Golgi (62)) (61,63). Human islet cells, on the other hand, are relatively insensitive to CPA, but show particular susceptibility to Brefeldin A, which efficiently induces ER stress and apoptosis in these cells ((61); Igoillo-Esteve M et al., unpublished data). Brefeldin A is also the only ER stressor able to induce apoptosis in the human cell line EndoC-βH1 after 24 h of exposure (Igoillo-Esteve M et al., unpublished data), while they are relatively insensitive to thapsigargin (57). The low sensitivity shown by both human islet cells and EndoC-βH1 cells to the SERCA-2b blocker CPA suggests the presence of other ER Ca^2+^ pumps that regulate ER Ca^2+^ homeostasis in human beta cells.

**Figure 2.**
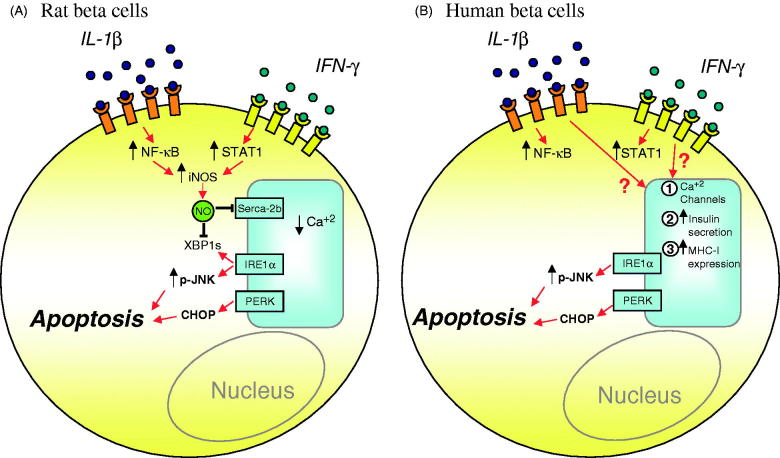
Proposed models for ER stress-induced apoptosis in cytokine-treated rat and human beta cells. A: IL-1β + IFN-γ, via NO production and consequent SERCA-2b inhibition, cause severe ER Ca^2+^ depletion in rat beta cells. NO also inhibits XBP1s protein expression, depriving these cells of a relevant adaptive mechanism. B: In human beta cells IL-1β + IFN-γ induce ER stress via mechanisms that are independent of NO production. The nature of these mechanisms remains to be clarified, but they may be related to inhibition of other Ca^2+^ channels, excessive insulin secretion, and over-expression of MHC class I and related proteins. The persistent activation of the IRE1α/JNK and PERK/CHOP pathways contributes to apoptosis in both rat and human beta cells.

**Figure 3.**
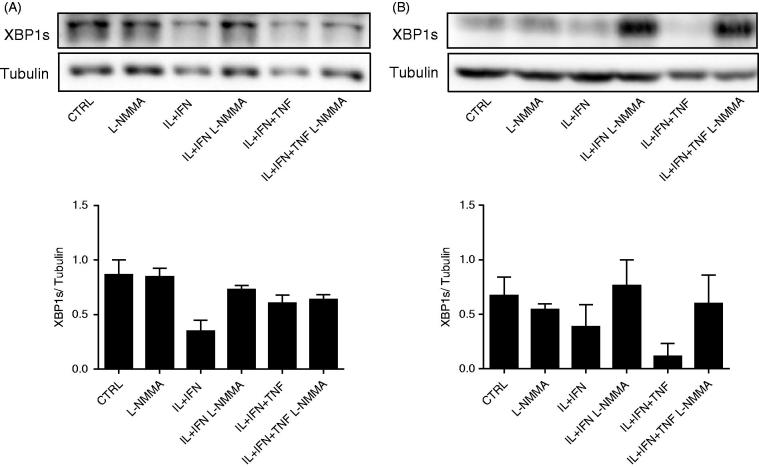
Cytokine-induced decrease in XBP1s protein expression is dependent on NO production in INS-1E and MIN6 cells. INS-1E cells (A) and MIN6 cells (B) were left untreated (CTRL) or treated with cytokines (as indicated), alone or in combination with 1 mmol/L of L-NMMA, for 24 h. A representative blot for XBP1s and tubulin, and the densitometric measurement of *n* = 3–4 experiments are shown. The Western blot data were normalized by the highest value considered as 1.

Regarding cytokines it was initially shown, in rat beta cells and INS-1E cells, that the combination of interleukin (IL)-1β and interferon (IFN)-γ causes severe ER Ca^2+^ decrease, UPR induction, and apoptosis via inhibition of SERCA-2b secondary to NO production (Figure 2A) (64). On the other hand, they down-regulate SERCA-2b and induce XBP1 splicing and PERK/eIF2α phosphorylation in mouse islets and MIN6 cells to a large extent independently of NO production (65). These data confirmed previous findings on inducible nitric oxide synthase (iNOS) knock-out (KO) mice, where cytokine-induced apoptosis was mostly NO-independent, whereas necrosis required NO formation (66). Interestingly, prolonged exposure to IL-1β + IFN-γ increases XBP1s mRNA (41,64), but decreases XBP1s protein expression in both INS-1E and MIN6 cells (Figure 3), probably contributing to the triggering of apoptosis (see above). The inhibition of NO formation by L-N^G^-monomethyl Arginine (L-NMMA) restores XBP1s protein expression in cytokine-treated rat and mouse cell lines (Figure 3), suggesting an additional role of cytokine-induced NO in the regulation of UPR.

In human islets TNF-α + IFN-γ cause a more marked activation of the ER stress response (67) in spite of the fact that IL-1β + IFN-γ induce a nearly 10-fold higher increase in NO production (68,69). Human islets are more resistant to the suppressive effects of cytokines than isolated rodent islets, and cytokine-induced human islet dysfunction is not prevented by iNOS inhibitors, suggesting that NO is not a major mediator of the deleterious effects of cytokines on human islets (68). We recently confirmed that inhibition of NO formation does not prevent cytokine-induced ER stress activation and apoptosis in human islet cells and in the human beta cell line EndoC-βH1 (41). Moreover, the expression of SERCA-2b is not modified by cytokines in human islets and in human insulin-producing EndoC-βH1 cells (41,70). Importantly, EndoC-βH1 cells do not express iNOS after cytokine treatment, despite the fact that cytokines induce ER stress and apoptosis in these cells (41). In line with these findings, previous studies have suggested that the main source of NO production by human islets are the ductal cells (71) and not the beta cells themselves (72). Taken together, these observations suggest that NO and consequent SERCA-2b inhibition are not major players for the induction of ER stress in human beta cells, and that other pathway(s) lead to cytokine-induced ER stress in rodents and human beta cells (41). The nature of these pathways remains unclear, but they may be related to the early and pronounced cytokine-induced increase in insulin release (68), effects on other Ca^2+^ pumps besides SERCA-2b, massive up-regulation of human major histocompatibility complex (MHC) class I expression, and perhaps delayed pro-insulin processing (Figure 2B).

Cytokines induce an IRE1α-dependent and biphasic JNK activation in human EndoC-βH1 cells, with biphasic peaks at 0.5 and 8 h. The KD of IRE1α with three independent small interfering RNAs (siRNAs) decreased JNK phosphorylation by ∼40% in EndoC-βH1 cells after both 0.5 and 8 h of IL-1β + IFN-γ treatment, indicating that the IRE1α pathway contributes to both early and late JNK activation in cytokine-exposed human beta cells (41). As discussed above, JNK has an important role for cytokine-induced human and rat beta cell apoptosis, in spite of the fact that the early events for the induction of ER stress are different between these two species. Indeed, suppression of JNK1 expression with two independent siRNAs partially protected human beta cells against cytokine-induced apoptosis (41). Interestingly, the chemical chaperone TUDCA, previously shown to protect NOD mice against the development of diabetes (32), significantly decreases cytokine-induced JNK phosphorylation and protects against cytokine-induced human beta cell apoptosis (41).

Of interest is the very early activation of IRE1α/JNK in human beta cells. This takes place after only 0.5 h of exposure to pro-inflammatory cytokines, suggesting an activation of this specific UPR pathway before (and therefore independently of) severe ER stress, which takes place after 6–8 h. The cross-talk between plasma membrane signaling and UPR components has been described in different cellular systems. Thus, IRE1α/XBP1s are activated by Toll-like receptors in macrophages, CD40 signaling in hepatocytes, vascular endothelial growth factor (VEGF)/PLCγ in endothelial cells, and B-cell receptors during plasma cell differentiation (49). It is thus conceivable that the early activation of the IRE1α/JNK pathway in cytokine-treated human beta cells occurs through direct activation by IL-1β and/or IFN-γ receptors, preceding the actual ER stress.

ER stress-independent functions of the UPR have been identified in beta cells. As described above, IRE1α has been proposed to monitor fluctuations in glucose levels in beta cells in the absence of ER stress, an effect mediated by its phosphorylation which occurs independently of the release of BiP from the luminal domain (52). Moreover, a recent study using inducible and beta cell-specific IRE1α deletion in mice demonstrated that the IRE1α/XBP1s pathway is essential for glucose-stimulated insulin biogenesis in mature beta cells (5). This IRE1α-dependent pathway was shown to regulate proinsulin mRNA translation, ribosome recruitment and structure, signal peptide cleavage, and suppression of oxidative/inflammatory stress. In this case, the early activation of IRE1α again seems to occur independently of ER stress and before the massive induction of insulin biosynthesis by glucose stimulation (5).

## Concluding remarks

The UPR is a conserved response, activated to restore proteostasis when cells accumulate unfolded proteins in the ER. Emerging evidence indicates new beta cell-specific functions of the IRE1α pathway, independently of ER stress activation, suggesting that UPR sensors have evolved to fulfill specific requirements in different cell types.

In case of unresolved and severe ER stress, persistent activation of the UPR triggers apoptosis. This is probably one of the mechanisms by which pro-inflammatory cytokines induce apoptosis in pancreatic beta cells during type 1 diabetes (T1D). JNK represents a major IRE1α-regulated pathway leading to beta cell apoptosis. The pathophysiological relevance of this finding is reinforced by the above-described discovery of two endogenous proteins that provide a specific negative feedback against IRE1α-induced JNK activation in beta cells (40) (Brozzi F et al., submitted for publication).

The challenge is now to understand the ultimate mechanisms by which pro-inflammatory cytokines induce IRE1α and JNK activation in human beta cells and, based on this information, to develop new and specific agents that prevent the pro-apoptotic signals of the UPR without affecting its homeostatic roles. These agents, together with re-education of the immune system, may contribute to prevent the aggravation of inflammation and progressive beta cell death in the transition between autoantibody positivity and actual T1D.

## Declaration of interest

The authors report no conflicts of interest.

Work by the authors was supported by grants from the Juvenile Diabetes Research Foundation International (JDRF grant number 17-2013-515), European Union (projects NAMIT and BetaBat, in the Framework Programme 7 of the European Community), Actions de Recherche Concertée de la Communauté Française (ARC), and the Fonds National de la Recherche Scientifique (FNRS), Belgium.
